# The benefit of specialized team approaches in patients with acute kidney injury undergoing continuous renal replacement therapy: propensity score matched analysis

**DOI:** 10.1186/s13054-014-0454-8

**Published:** 2014-08-13

**Authors:** Hyung Jung Oh, Mi Jung Lee, Chan Ho Kim, Dae Young Kim, Hye Sun Lee, Jung Tak Park, Sungwon Na, Seung Hyeok Han, Shin-Wook Kang, Shin Ok Koh, Tae-Hyun Yoo

**Affiliations:** Department of Internal Medicine, College of Medicine, Yonsei University, 50-1 Yonsei-ro, Seodaemoon-Gu, Seoul, 120-752 Korea; Yonsei University Health System, Severance Hospital, Seoul, Korea; Department of Biostatistics, Yonsei University College of Medicine, Seoul, Korea; Department of Anesthesiology and Pain Medicine, Yonsei University College of Medicine, Seoul, Korea

## Abstract

**Introduction:**

Continuous renal replacement therapy (CRRT) has been widely used in critically ill acute kidney injury (AKI) patients. Moreover, some centers operate a specialized CRRT team (SCT) composed of physicians and nurses, but few studies have yet determined the superiority of SCT control.

**Methods:**

A total of 334 among 534 patients in the original cohort, who started CRRT for severe AKI between August 2007 and September 2009 in Yonsei University Health System and were matched with a propensity score (PS), were divided into two groups based on SCT application. Moreover, we compared CRRT-related outcomes including down-time per day and lost time per filter-exchange between the two groups. The primary outcomes were 28- and 90-day all-cause mortality, and the secondary outcomes were the rates of renal function recovery at 28- and 90-day.

**Results:**

The down-time per day, lost time per filter-exchange, and red blood cell-transfused numbers during CRRT treatment were significantly lower after SCT approach compared with the group before SCT, while net ultrafiltration rate in the after SCT group was significantly higher compared to the before SCT group. During the study period, the 28- and 90-day all-cause mortality rates were significantly decreased after SCT application. Cox regression analysis revealed that 28- and 90-day all-cause mortality rates were significantly lower under SCT control, after adjusting for primary diagnosis, emergent surgical cases, Charlson Comorbidity Index and biochemical parameters. However, there were no significant differences in the rate of renal function recovery before and after SCT approach in CRRT.

**Conclusions:**

A well-organized CRRT team could be beneficial for clinical outcomes through improving quality of care in AKI patients requiring CRRT treatment in the ICU.

**Electronic supplementary material:**

The online version of this article (doi:10.1186/s13054-014-0454-8) contains supplementary material, which is available to authorized users.

## Introduction

Severe acute kidney injury (AKI) is a well-recognized complication in critically ill patients and has a substantial impact on morbidity, mortality, and health resource utilization in this population [1-5]. Although only conservative treatment such as fluid and hemodynamic optimization was provided for critically ill patients with severe AKI in the past [6], continuous renal replacement therapy (CRRT) has recently been an integral part of critical care and is considered an established treatment modality for AKI patients [7]. Even though recent advances in technical devices have widened clinical indications for CRRT, the mortality rate in this population still remains extremely high [8-10]. Given the complexity of treating AKI patients and handling the extracorporeal system, highly efficient CRRT management, which includes proper exchange of extracorporeal circuits, frequent monitoring for dose of CRRT, and optimal anticoagulation and replacement of electrolytes, are regarded as potential candidates for improving patient outcomes [9,11].

It has been speculated that repeated quality control is essential to obtain optimal management of CRRT. Therefore, some centers operate specialized CRRT teams (SCT) with physicians and nurses from their disciplines [12]. It might be suggested that the survival rates of the patients after care by the SCT would be superior; however, to our knowledge, only one study has been reported on the comparison before and after the SCT approach [12]. Moreover, several factors, including patients’ severity scores, make it difficult to clarify the benefit of SCT management. We initiated the SCT approach for the management of CRRT in 2008, and thus we can compare the outcomes and quality of CRRT management before and after the SCT approach. In addition, we used propensity score (PS) matching to investigate the benefit of SCT management for 28- and 90-day all-cause mortalities and renal function recovery in AKI patients undergoing CRRT.

## Methods

### Patients

**Figure 1:**
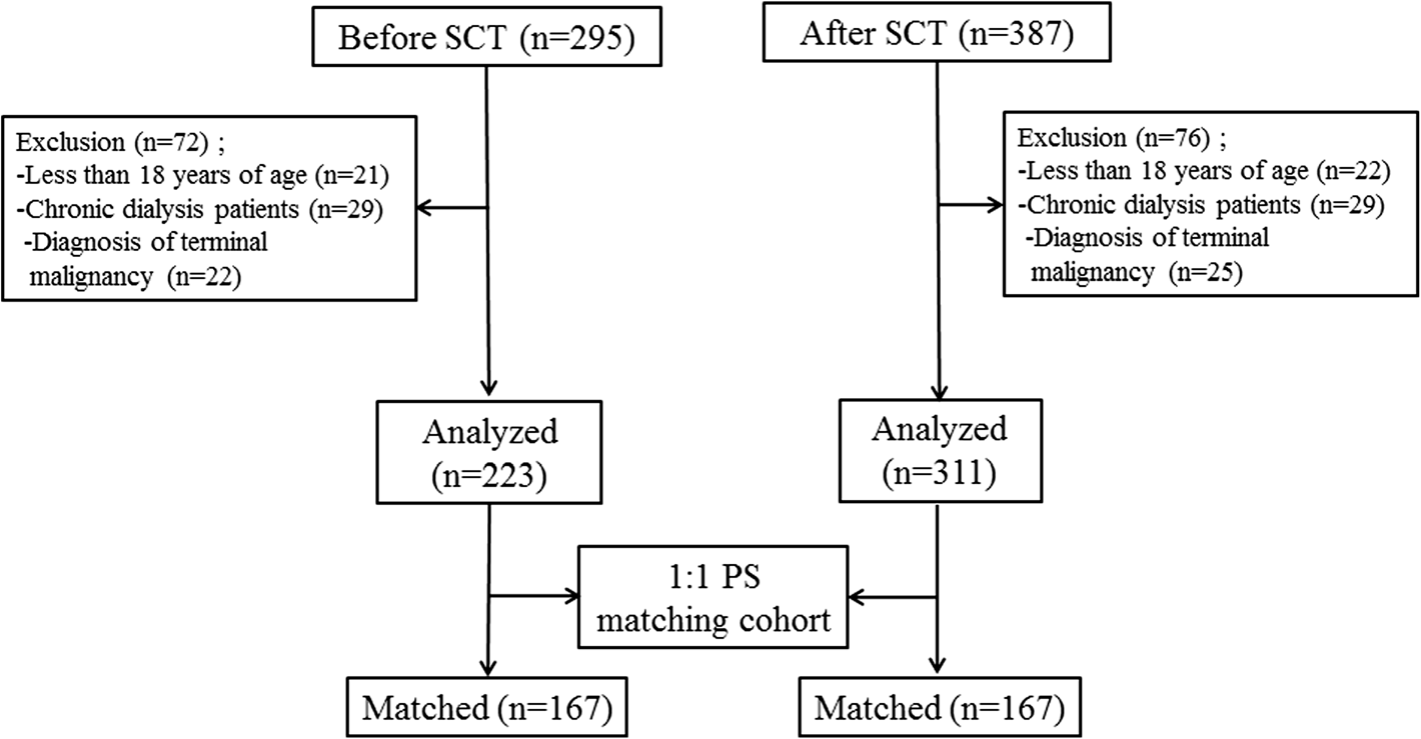
**Flow diagram of patient selection and outcomes.** From August 2007 through September 2009, we enrolled 295 and 387 patients in the groups before and after SCT, respectively. After 1:1 propensity score matching, each of the 167 patients before and after the SCT approach was ultimately analyzed. SCT, specialized continuous renal replacement therapy team; CRRT, continuous renal replacement therapy; PS, propensity score.

A total of 682 patients who started CRRT for severe AKI between August 2007 and September 2009 were initially analyzed. We excluded 148 patients because they were below 18 years of age, were on chronic dialysis, or were diagnosed with terminal malignancy with less than 3 months of life expectancy. Therefore, 534 patients were included in the final analysis (Figure 1).

The study protocol was approved by the Institutional Review Board (IRB) of the Yonsei University Health System (YUHS) Clinical Trial Center. As this study was a retrospective medical record-based study and the study subjects were de-identified, the IRB waived the need for written consent from the patients.

### Data collection

Patients’ data were retrieved from the CRRT Database of YUHS, Seoul, Korea. Demographic, clinical, and biochemical data at the time of admission to the ICU and CRRT initiation were recorded. For the assessment of disease severity, the sequential organ failure assessment (SOFA) score and acute physiology and chronic health evaluation (APACHE) II score were determined at the start of CRRT.

We counted the transfused number of packed red blood cells (RBC) during the period of CRRT treatment except for transfusions conducted due to active bleeding. Such active bleeding is considered to be in a situation where the patients need a transfusion of more than 10 units of packed RBC within 24 hrs or are bleeding more than 1- to 1.5-fold of the body’s entire blood volume as previously described [13]. Down-time (hours/day) was defined as the period of time when CRRT was not applied between initiation and end of CRRT, as defined by Uchino [14].

### SCT approach

**Figure 2:**
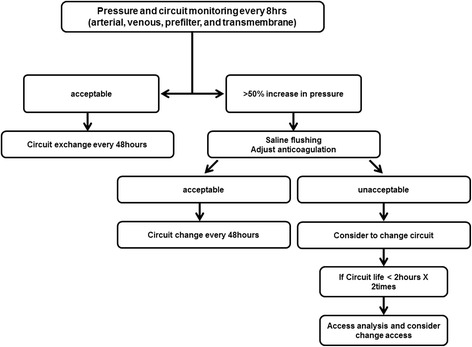
**Flow diagram of programmed SCT management of vascular access.** When CRRT starts, SCT monitors circuit pressure (arterial, venous, and transmembrane) every 8 hrs. Extracorporeal circuit exchanges are conducted according to appointed protocols for access management. SCT, specialized continuous renal replacement therapy team; CRRT, continuous renal replacement therapy.

An SCT was set up in August 2008 in our hospital, and thus we can compare clinical outcomes before and after the SCT approach. The SCT is defined as a team of physicians and nurses, who are specially trained and educated to perform CRRT. The members of the SCT include two specialized nephrologists, two nephrology fellows, an ICU specialist, three ICU residents, and five CRRT specialized nurses. They create and share the educational programs and management protocol on CRRT. In addition, monthly quality control is performed to correct management protocols and problems with CRRT, such as electrolytes, coagulation, and hemodynamic status. Moreover, we have adopted and used the anticoagulation protocol, which was suggested from Bagshaw *et al*. [15]. Members of the SCT team have complementary roles to each other. ICU specialist and residents have a primary responsibility for general patient care and make overall decisions about the medical problems of ICU patients. Especially, nephrologists are authorized to initiate, maintain and stop CRRT and switch to intermittent hemodialysis or peritoneal dialysis. They decide whether to start CRRT based on the patient’s hemodynamic status, declining urine output, and electrolyte and acid-base imbalances and also decide the mode and dose of CRRT, and net ultrafiltration rate during CRRT. In the nursing section, CRRT-nurses work three shifts daily. They monitor the patients treated with CRRT on the ICU at regular intervals, and check hemodynamic stability, the removal rate of real ultrafiltration, and the status of the CRRT kit, et cetera. Their primary role is CRRT management apart from the ICU general care, which is performed by bedside nurses. In addition, they also provide education for basic CRRT-handling methods to bedside nurses every three months. Half of the CRRT nurses rotate among the ICU nurses every year. Based on daily rounds with the specialized team, they discuss and try to solve potential problems with management of CRRT (Figure 2). Additional tables show these descriptions in more detail (See Additional file 1: Tables 1 and 2).

### ICU setting

The investigation site was a self-contained, 112-bed medical and surgical ICU in a 2,089-bed teaching hospital in Seoul, Korea that was equipped with 15 CRRT machines. The ICU unit is a closed structure within this hospital. There was no difference in the determination of CRRT treatment according to decisions made by the same nephrologist before and after SCT. On the contrary, bedside nurses cared for their ICU patients at a ratio of two patients per nurse, but they also had to perform monitoring and maintenance of the CRRT system before SCT set-up. However, after the introduction of the SCT approach, additional nursing care was available for CRRT care.

### CRRT protocol

Vascular access for CRRT was obtained via the internal jugular, femoral, or subclavian vein. In most patients, continuous veno-venous hemodiafiltration, including slow continuous ultrafiltration, was performed using the PRISMA (Gambro, Hechingen, Germany) platform. CRRT was initiated at a blood flow rate of 100 mL/minute, which was gradually increased to 150 mL/minute. The ultrafiltration dose was set at 35 mL/kg/hr, and Hemosol® (Gambro) was replaced by the predilution method. We also measured the delivered drainage amount and recorded it to calculate the delivered CRRT dose daily. Circuit exchanges were conducted regularly after 48 hrs of use, even if the blood pumps were not stopped.

### Statistical analysis

This study was a matched cohort study using two groups of patients before and after SCT management to analyze the effect of several potential confounders. The patients were matched 1:1 by PS using the greedy matching algorithm (eight- to one-digit match) [16]. Once the group of patients before SCT was matched, the patients after SCT were not reconsidered. The algorithm made the *best* matches first and *next-best* matches second in a hierarchical sequence until no additional matches could be made. Best matches were those with the highest digit match on PS. First, patients in the group before SCT were matched to patients in the group after SCT on eight digits of the PS. For those that did not match, patients from the group before SCT were then matched to patients after SCT based on seven digits of the PS. The algorithm proceeded sequentially to the lowest digit match on PS (one digit). We derived the PS from a multi-logistical regression model with the following variables: age, gender, mean arterial pressure (MAP), SOFA and APACHE II scores, and risk, injury, failure, loss, and end kidney disease (RIFLE) criteria. After all PS matches were performed, we assessed the balance in baseline covariates between the two groups using the Wilcoxon signed rank test for continuous variables and the McNemar test for categorical variables. PS matching was conducted using SAS version 9.1 (SAS Institute, Cary, NC, USA). We also presented original data before PS matching and compared clinical outcomes between the before-SCT and after-SCT groups in both original and matched cohorts, respectively.

Continuous variables are presented as mean ± SD and categorical variables as numbers and percentages, if they were statically normally distributed. Moreover, they were compared using the Student *t*-test for continuous variables and the χ^2^ test for categorical variables. However, medians and interquartile ranges are presented unless variables are clearly normally distributed, and they were compared using Mann-Whitney test. In the present study, we evaluated 28- and 90-day all-cause mortalities as primary endpoints. We also compared the proportion of patients with renal function recovery, which was defined based on creatinine clearance (≥15 mL/minute) with no need of renal replacement therapy at 28- and 90-day intervals, between the groups, as secondary endpoints.

Survival curves were designed using the Kaplan-Meier method, and comparisons were made using the log-rank test. The impact of SCT control on 28- and 90-day mortalities in AKI patients treated with CRRT was determined using the Cox proportional hazards model, and the results are presented as hazard ratios (HRs) and 95% CIs. To confirm the assumption of proportionality, time-dependent covariate analysis was used, and it was not statistically significant, suggesting that the proportional hazards assumption was reasonable. All tests were two-sided, and *P* <0.05 was considered significant. Statistical analyses were performed with SPSS for Windows, version 18.0 (SPSS Inc., Chicago, IL, USA).

## Results

### Baseline characteristics in both original and matched groups

Baseline characteristics of these patients are shown in Table 1. In the original cohort, the proportion of male patients was significantly higher in the after-SCT group compared with the before-SCT group (68.2% versus 58.7%, *P* = 0.028), while APACHE II score and SOFA score were significantly lower in the after-SCT group compared to the before-SCT group (APACHE II score; 26.9 versus 28.4, *P* = 0.029 and SOFA score; 11.9 versus 14.2, *P* = 0.031). However, there were no significant differences in age, MAP, age-adjusted CCI, RIFLE status, contributing factors for AKI, primary diagnosis, emergent surgical cases, use of anticoagulation and diuretics, or biochemical parameters, between the two groups. The 1:1 PS matching yielded matched pairs of 167 patients in the before-SCT group and 167 patients in the after-SCT group, resulting in no differences in the above-mentioned variables.

**Table 1:** **Baseline characteristics at CRRT initiation in the original and the matched cohort**

**Variables**	**Original cohort**		***P*** **-value**	**Matched cohort**		***P*** **-value**
	**Before SCT (n = 223)**	**After SCT (n = 311)**		**Before SCT (n = 167)**	**After SCT (n = 167)**	
Age, years	62.8 ± 14.0	61.6 ± 15.1	0.350	63.2 ± 13.6	63.1 ± 15.1	0.961
Male, n (%)	131 (58.7%)	212 (68.2%)	0.028	99 (59.3%)	99 (59.3%)	>0.999
Mean arterial pressure, mmHg	80.7 ± 17.3	78.7 ± 16.0	0.168	80.7 ± 17.5	79.9 ± 15.2	0.881
APACHE II score	28.4 ± 8.6	26.1 ± 7.0	0.029	27.6 ± 8.1	27.4 ± 6.9	0.783
Sequential organ failure assessment score	14.2 ± 3.9	11.9 ± 3.4	0.031	12.1 ± 2.8	12.1 ± 3.3	0.958
Age-adjusted Charlson comorbidity index	6.1 ± 3.5	5.7 ± 3.0	0.365	5.9 ± 3.1	5.7 ± 2.8	0.886
RIFLE, n (%)			0.647			0.901
Risk	96 (43.1%)	140 (45.0%)		75 (44.9%)	75 (44.9%)	
Injury	79 (35.4%)	108 (34.7%)		56 (33.5%)	59 (35.3%)	
Failure	48 (21.5%)	63 (20.3%)		36 (21.6%)	33 (19.8%)	
Contributing factors, n (%)			0.681			0.849
Sepsis	112 (50.2%)	158 (50.8%)		87 (52.1%)	88 (52.7%)	
Hemodynamic instability without sepsis	84 (37.7%)	106 (34.1%)		55 (32.9%)	51 (30.5%)	
Major surgery	27 (12.1%)	47 (15.1%)		25 (15.0%)	28 (16.8%)	
Use of anticoagulation, n (%)	179 (80.3%)	235 (75.6%)	0.209	129 (77.2%)	128 (76.6%)	0.891
Diuretics use, n (%)	178 (79.8%)	246 (79.1%)	0.831	129 (77.2%)	128 (76.6%)	0.787
Biochemical data						
Hemoglobin, g/L	92 ± 18	92 ± 18	0.987	92 ± 18	92 ± 19	0.837
Whole blood cells, 10^3^/mm^3^	15.6 ± 12.2	14.1 ± 10.5	0.174	15.2 ± 12.0	14.5 ± 11.0	0.577
Blood urea nitrogen, mmol/L	20.2 ± 9.5	19.0 ± 10.4	0.166	20.7 ± 9.8	18.7 ± 10.0	0.066
Creatinine, umol/L	300.6 ± 167.9	309.4 ± 203.3	0.681	318.2 ± 176.8	300.6 ± 185.6	0.393
Total cholesterol, mmol/L	95.2 ± 45.5	92.3 ± 43.4	0.495	95.5 ± 45.9	91.6 ± 41.3	0.411
Albumin, g/L	26 ± 6	27 ± 6	0.209	26 ± 6	28 ± 6	0.106
C-reactive protein, mg/L	14.3 ± 12.0	12.4 ± 12.7	0.106	14.4 ± 11.9	12.8 ± 11.2	0.300
Arterial pH	7.4 ± 0.1	7.3 ± 0.4	0.174	7.3 ± 0.1	7.3 ± 0.5	0.080
Total bilirubin, umol/L	68.4 ± 123.1	63.3 ± 109.4	0.602	66.7 ± 119.7	63.3 ± 100.9	0.814
HCO_3_^−^, mmol/L	19.9 ± 4.9	19.2 ± 5.5	0.157	20.0 ± 4.8	19.1 ± 5.7	0.125

Data are presented as n (%) or mean ± SD. CRRT, continuous renal replacement therapy; SCT, specialized CRRT team; APACHE II, acute physiology and chronic health evaluation II; RIFLE, risk, injury, failure, loss, and end kidney disease.

### Comparisons of clinical outcomes and renal outcomes in original and matched groups during follow up

The down-time per day, lost time per filter-exchange, RBC-transfused numbers, and the transfusion rates during CRRT treatment were significantly lower after the SCT approach compared to in group before SCT in both cohorts, while net ultrafiltration rate in the after-SCT group was significantly higher compared to that in the before-SCT group. However, total CRRT time was significantly lower under SCT in both cohorts, whereas the median filter lifespan during the CRRT treatment was significantly higher after the SCT approach compared with the group before SCT only in original cohort (Table 2). On the contrary, there were no significant differences in the proportions of living patients with renal function recovery at 28-day and 90-day intervals between the two groups in both original and matched cohorts (Table 3). Moreover, vasoactive drugs such as dopamine and norepinephrine were required in 390 patients (73.0%), who were 166 patients (74.4%) in the before-SCT group and 224 patients (72.0%) in the SCT group before CRRT start. Ten days after CRRT application, 21 patients of 83 survivors in the before-SCT group and 26 patients of 106 survivors in the after-SCT group continuously received vasoactive drugs, and there was no significant difference between the two in the use of vasoactive medications.

**Table 2:** **Study outcomes and parameters associated with CRRT in groups of patients before and after SCT in both cohorts**

	**Unmatched cohort**		***P*** **-value**	**Matched cohort**		***P*** **-value**
	**Before SCT (n = 223)**	**After SCT (n = 311)**		**Before SCT (n = 167)**	**After SCT (n = 167)**	
Total CRRT time, days	7 (1 to 48)	4 (1 to 34)	0.023	5 (1 to 48)	4 (1 to 32)	0.197
Down-time per day, hrs	5.2 (3.7-16.5)	3.1 (2.5 to 5.9)	<0.001	4.8 (3.7 to 9.4)	3.3 (2.8 to 5.7)	<0.001
Lost time per filter-exchange, minutes	43 (28 to 57)	25 (20 to 32)	<0.001	42 (31 to 55)	23 (20 to 30)	<0.001
Ultrafiltration rate, mL/kg/hr	22.5 (19.8 to 28.1)	27.1 (25.1 to 30.5)	0.031	24.5 (22.5 to 28.1)	28.2 (26.3 to 30.5)	0.039
Number of TF during CRRT	9 (1 to 22)	7 (1 to 17)	0.028	8 (1 to 22)	6 (1 to 14)	0.021
TF rate, n (%)	165 (74.0%)	208 (66.9%)	0.039	118 (70.7%)	106 (63.5%)	0.043
Filter life span, hrs	22.0 (5.9 to 44.1)	29.4 (6.1 to 40.1)	<0.001	25.7 (5.9 to 43.8)	31.1 (6.1 to 39.7)	0.084
CRRT mortality, n (%)						
28 days	154 (69.1%)	155 (49.8%)	0.013	104 (62.3%)	81 (48.5%)	0.015
90 days	170 (76.2%)	187 (60.1%)	0.026	118 (70.7%)	99 (59.3%)	0.039

Data are presented as n (%) and median and interquartile ranges. CRRT, continuous renal replacement therapy; SCT, specialized CRRT team; TF, transfusions; ICU, intensive care unit.

**Table 3:** **Comparison of renal function recovery among survivors in the original and matched cohorts**

**Variables**	**Original cohort**		***P*** **-value**	**Matched cohort**		***P*** **-value**
	**Before SCT**	**After SCT**		**Before SCT**	**After SCT**	
Recovery of renal function, n (%)^*^						
28 days	47/69 (68.1%)	109/156 (69.9%)	0.663	42/63 (66.7%)	60/86 (69.8%)	0.592
90 days	47/53 (88.7%)	111/124 (89.5%)	0.779	44/49 (89.8%)	61/68 (89.7%)	0.866

Data are presented as number/total number (%). SCT, specialized continuous renal replacement therapy team. ^*^Recovery of renal function was defined based on creatinine clearance (≥15 mL/minute) with no need for renal replacement therapy.

### SCT approach is independently associated with survival in AKI patients

**Figure 3:**
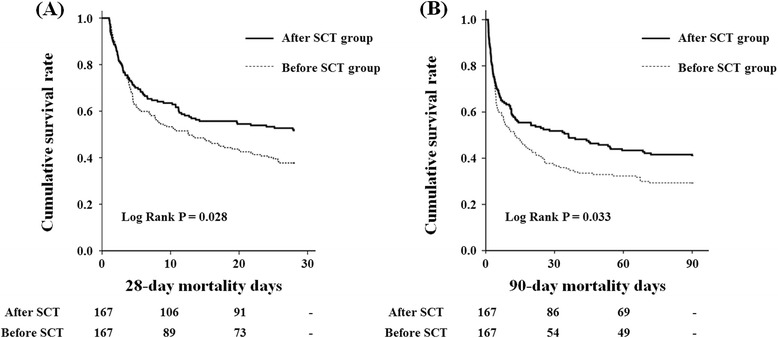
**Kaplan-Meier plots for cumulative 28- and 90-day mortality.** The 28- and 90-day all-cause mortality rates after the SCT approach were significantly lower; log rank *P* = 0.028 **(A)** and *P* = 0.033 in **(B)**. SCT, specialized continuous renal replacement therapy team.

In the original cohort, 28- and 90-day all-cause mortality was significantly higher in the group before SCT compared to in the group after SCT (69.1% versus 49.8%, *P* = 0.013 and 76.2% versus 60.1%, *P* = 0.026, respectively). These findings were also consistent in the matched cohort (before-SCT group versus after-SCT group; 62.3% versus 48.5%, *P* = 0.015 for 28-day all-cause mortality, and 70.7% versus 59.3%, *P* = 0.039 for 90-day all-cause mortality, respectively). In addition, Kaplan Meier plots demonstrated that 28- and 90-day all-cause mortality was significantly higher in the group before SCT (*P* = 0.028 for 28-day and *P* = 0.033 for 90-day mortality, respectively) (Figure 3). Cox regression analysis revealed that 28- and 90-day all-cause mortality rates were significantly reduced after SCT (HR 0.643; 95% CI 0.470, 0.879; *P* = 0.006 for 28-day and HR 0.680; 95% CI 0.510, 0.906; P = 0.008 for 90-day mortality) (model 1). Moreover, the lower mortality risk in the group on SCT remained significant even after adjusting for model 1 plus Hb, serum albumin, total cholesterol, and C-reactive protein (CRP) levels (HR 0.720; 95% CI 0.540, 0.968; *P* = 0.027 for 28-day and HR 0.742; 95% CI 0.570, 0.988; *P* = 0.039 for 90-day mortality) (model 2). Furthermore, adjustments for model 2 plus primary diagnosis, emergent surgical cases and age-adjusted CCI did not change the benefit for primary outcomes of the SCT approach (HR 0.897; 95% CI 0.681, 0.982; *P* = 0.040 for 28-day mortality and HR 0.927; 95% CI 0.725, 0.997; *P* = 0.042 for 90-day mortality) (model 3) (Table 4).

**Table 4:** **Cox proportional hazards models for 28- and 90-day mortality in the SCT group**

	**Compared with before SCT management**					
	**Mortality at 28 days**			**Mortality at 90 days**		
	**HR**	**95% CI**	***P*** **-value**	**HR**	**95% CI**	***P*** **-value**
Model 1	0.643	0.470, 0.879	0.006	0.680	0.510, 0.906	0.008
Model 2	0.720	0.540, 0.968	0.027	0.742	0.570, 0.988	0.039
Model 3	0.897	0.681, 0.982	0.040	0.927	0.725, 0.997	0.042

Model 1: unadjusted hazard ratio; model 2: Model 1 with additional adjustments for hemoglobin, serum albumin, total cholesterol, and C-reactive protein levels; Model 3: model 3 with primary diagnosis, emergent surgical procedure and age-adjusted Charlson comorbidity index. CRRT, continuous renal replacement therapy; SCT, specialized CRRT team; HR, hazard ratio.

## Discussion

In this study, we found that the SCT approach for managing CRRT reduced 28- and 90-day mortality rates in AKI patients. These findings suggest that well-trained and well-organized team approaches have a beneficial effect on clinical outcomes in AKI patients treated with CRRT in the ICU.

CRRT has been preferred for AKI patients with hemodynamic instability to control uremia, electrolytes, acid-base status, and volume balance in many ICUs [9]. Although there is still controversy about the optimal dose of CRRT, the minimal required dose is necessary for adequate solute control and the correction of electrolyte and acid-base imbalance; however, frequent blood-pump halting and prolonged manipulation time for the replacement of tubing systems can result in inadequate treatment doses and blood loss in these patients [17,18]. Uchino *et al*. [14] reported that the median down-time was 3.0 hrs (1.0 to 8.3), thus, concluded that the term, continuous, in CRRT is somewhat inaccurate due to frequent interruptions in CRRT treatment. They also suggested that down-time adversely affected azotemic control, and therefore physicians who prescribe CRRT should be aware of the consequences of such down-time on the quality and quantity of renal replacement therapy [14]. A previous clinical trial, including a multinational observational study by Vesconi *et al*. [19], also showed a wide variability in ultrafiltration rates with large differences between the prescribed and delivered doses, which was mainly due to circuit down-time [14,20-22]. The patients treated with CRRT are essentially immobile, frequently have to be sedated, and can develop hypothermia [23]. Moreover, a large number of patients have disseminated intravascular coagulation. Therefore, extracorporeal circuits are prone to clotting in most of the patients [23]. Apart from clotting in the extracorporeal circulation, vascular access problems frequently occur. Taken together, access problems and thrombogenecity reduce the circuit lifetime in this population [23]. Frequent interruptions of CRRT due to extracorporeal circuit failure inevitably increase CRRT down-time and are also associated with blood loss, requiring multiple blood transfusions [24], and increasing costs [23]. Especially, AKI patients requiring CRRT treatment are mostly susceptible to fluid balance. If the longer CRRT down-time persists, the more fluid removal will be needed during the remaining time in order to overcome volume overload. Fast removal of fluid by net ultrafiltration may lead to sympathetic over-activity and harmful effects on the myocardium, and increase the risk of hypoperfusion to vital organs [25,26].

In this study, the down-time per day, lost time per filter-exchange, and numbers of RBC transfused during CRRT were significantly reduced after the SCT approach compared with the group before SCT. In addition, the 28- and 90-day mortality rates in the after-SCT group were significantly lower compared with the before-SCT group. Taken together, we surmise that decreased down-times, lost time per filter-exchange, and numbers of RBC transfused should play indispensable roles to reduce the mortality in AKI patients requiring CRRT.

Still, the gaps in down-time and RBC transfusion requirements are not enough to explain the difference in CRRT mortality between the two groups. Gilbert *et al*.[12] demonstrated that the CRRT program definitely enhanced the level of care given to patients by allowing the bedside nurse on general patient care into the ICU. At the same time, the trained CRRT-nurse could concentrate solely on CRRT, thereby providing an additional level of care. For these reasons, they demonstrated that mortality rates in the CRRT program might be lower than expected, especially in critically ill burn patients [12]. Taken together, we suggest that the beneficial effects of the SCT approach could result from adequate application of management protocols, appropriate monitoring and exchange of CRRT, and frequent assessment and optimal troubleshooting in CRRT management.

Our study has several potential limitations that should be noted. First, this was an observational study in which the effect of confounding by indication could not be fully excluded. Some important baseline covariates were not distributed equally and many confounding factors, including the severity score, usually affect patient outcomes in this population. In the original cohort, the proportion of male patients was significantly lower in the before-SCT group than in the after-SCT group, whereas APACHE II and SOFA scores were significantly higher in the before-SCT group compared to the after-SCT group, which might lead to potential bias. Using PS matching, an identical, matched cohort at baseline between the two groups was created. However, a prospectively planned cluster or stepped wedge trial at multiple centers could be more helpful to evaluate the impact of an SCT in the future.

Second, the two groups before and after the SCT approach were not conducted at the same time. Therefore, there might be differences in the treatment trends during the study periods; however, the gap between the periods was only 1 year; also, the same ICU policy was applied to the patients in a single center, the patients in this study experienced no differences in ICU care, such as the decision-making process for CRRT, and there were few missing datasets. Moreover, as shown in Table 1, there were no significant differences in the baseline characteristics between the two groups after PS matching. Therefore, the bias caused by the different time periods may be overcome.

Third, the mortality rates of our patients were somewhat higher compared with those in previous studies of patients with AKI treated with CRRT [27]. However, Allegretti *et al*. [8] reported that in-hospital mortality was 61% for AKI patients requiring CRRT, and AKI with the same SOFA score contributed to a higher mortality rate. In addition, the severity of disease assessed by APACHE II and SOFA scores and age-adjusted CCI in this present study were somewhat higher compared with those of previous studies. Taken together, exceptionally high mortality rates could be expected, considering these patients had higher SOFA scores and needed CRRT. On the contrary, we examined the mortality rates at 28 and 90 days for all admissions to this ICU and for other common diagnoses (namely, sepsis, trauma, cardiac surgery, cardiovascular origin et cetera) during the follow-up period. With the exclusion of pediatric patients and patients who took one-day elective ICU admission after a procedure, survival rates during the follow up period were analyzed for a total of 6,222 patients. Sepsis in 1,901 patients (30.6%) was the most common diagnosis in the meantime. The 28- and 90-day all-cause mortality rates after SCT was set up were somewhat higher compared with those before SCT was established, but the differences were not statistically significant (*P* = 0.081 and 0.113, respectively). In the same way, there were no significant differences in 28- and 90-day mortality rates for each diagnosis between the two follow-up periods when we classified the patients according to common diagnoses (See Table 3 in Additional file 1). Despite these limitations, to our knowledge, this study may be the first report clarifying the impact of SCT control in the clinical outcomes of patients undergoing CRRT.

## Conclusions

A well-trained CRRT team could be beneficial for clinical outcomes through improving quality of care in AKI patients requiring CRRT in the ICU.

## Key messages

- A well-organized CRRT team could be beneficial for clinical outcomes through improving quality of care in AKI patients requiring CRRT in the ICU.
- This study aimed to compare the benefit of SCT by PS matching to overcome the differences from unbalanced distribution in the original cohort.
- The down-time per day, lost time per filter-exchange, and numbers of RBC transfused during CRRT were significantly lower after the SCT approach compared with the group before SCT, while the ultrafiltration rate in the after-SCT group was significantly higher compared to the before-SCT group. Moreover, the 28- and 90-day all-cause mortality rates were significantly decreased after SCT application.

## Additional file

Additional file 1: Table S1. The modules of the educational program for specialized continuous renal replacement therapy (CRRT) team (SCT) nurses. **Table S2.** The protocol for replacement of electrolytes and anticoagulation during CRRT. **Table S3.** The mortality rates at 28 and 90 days for all admissions to this ICU.

## Abbreviations

AKI: acute kidney injury
APACHE: acute physiology and chronic health evaluation
CCI: Charlson comorbidity index
CRP: C-reactive protein
CRRT: continuous renal replacement therapy
Hb: hemoglobin
HR: hazard ratio
MAP: mean arterial pressure
PS: propensity score
RBC: red blood cells
RIFLE: risk, injury, failure, loss, and end kidney disease
SCT: specialized continuous renal replacement therapy team
SOFA: sequential organ failure assessment
WBC: white blood cells
YUHS: Yonsei University Health System
