# Supplementary material for: The benefit of specialized team approaches in patients with acute kidney injury undergoing continuous renal replacement therapy: propensity score matched analysis
Source: Crit Care. 2014 Aug 13;18(4):454. doi: 10.1186/s13054-014-0454-8 (PMC4145553; doi:10.1186/s13054-014-0454-8)
Supplement: Additional file 1: Table S1. — The modules of the educational program for specialized continuous renal replacement therapy (CRRT) team (SCT) nurses. Table S2. The protocol for replacement of electrolytes and anticoagulation during CRRT. Table S3. The mortality rates at 28 and 90 days for all admissions to this ICU. [file 13054_2014_454_MOESM1_ESM.pdf]

Additional Table 1. The modules of educational program for SCT nurses

Module		Lessons	In-practice training
I	General intensive care course	72h	80h on general ICU
II	CRRT specialist training course	16h	32h on general ICU

Module I is corresponded to all ICU nurses. Module II is relevant to SCT nurses for CRRT.

*Module I includes several topics about the general management of AKI patients including nursing care for CRRT patients and practical installation and monitoring of the CRRT machine.

**CRRT specialist training course; Basic principles and optimal prescription for CRRT, Overview and practical operation of CRRT, trouble shooting in CRRT, assessment and management of failed dialysis catheter.

Additional Table 2. The protocol for replacement of electrolytes and anticoagulation during CRRT

Electrolytes	K^+ (mEq/L)	>4.5	No KCl mix in the 5L hemozol®
		3.6-4.5	20mEq KCl mix in the 5L hemozol®
		<3.6	40mEq KCl mix in the 5L hemozol®
	P (mEq/L)	≥2.0	No phosten® mix in the 5L hemozol®
		<2.0	20ml phosten® mix in the 5L hemozol®
Anticoagulation	Initial	High risk	No anticoagulation/saline flushing
		Low risk	Systemic heparinization
	Maintenance	High risk-1	No anticoagulation/saline flushing
		High risk-2	Regional anticoagulation (Citrate or Nafamostat)
		Low risk	Systemic heparinization

*Potassium and phosphate level check 2 times per day

*Phosten® ; potassium phosphate

*Hemosol® ; hemozol B0

*Definitions;

1) High risk; active bleeding, post-operative within 48hours, low platelet count <50,000/mm^3, prolonged PT/PTT ≥2.0 INR/60 sec

2) Low risk; all patients except for high risk patients

3) High risk-1 and high risk-2 were divided according to assessment of clotting in extracorporeal system during CRRT

Additional Table 3. The mortality rates at 28- and 90-day for all admissions to this ICU and for other common diagnoses

	28-day mortality			90-day mortality		
	Before SCT	After SCT	P-value	Before SCT	After SCT	P-value
Total, n(%)*	483/3,039 (15.9%)	573/3,183 (18.0%)	0.081	669/3,039 (22.0%)	797/3,183 (25.0%)	0.113
Sepsis	214/940 (22.8%)	257/961 (26.7%)	0.109	289/940 (30.7%)	329/961 (34.2%)	0.073
Trauma	68/457 (14.9%)	88/483 (18.2%)	0.137	94/457 (20.6%)	119/483 (24.6%)	0.072
Cardiac surgery	75/465 (16.1%)	78/455 (17.1%)	0.566	99/465 (21.3%)	115/455 (25.3%)	0.079
Cardiovascular origin**	104/759 (13.7%)	110/786 (14.0%)	0.881	137/759 (18.1%)	163/786 (20.7%)	0.097
Others	22/418 (5.3%)	40/498 (8.0%)	0.063	50/418 (12.0%)	71/498 (14.2%)	0.084

Abbreviations: ICU, intensive care unit

* With exclusion of pediatric patients and patients who took 1-day elective ICU admission after procedure, total of 6,222 patients was analyzed survival rates during follow up period.

** Any disease that affects the cardiovascular and cerebrovascular system
